# Surface-to-Bulk Elemental Profiling and Melissopalynological Characterization of Monofloral Bee Pollens: Implications for Nutritional Value and Health Risk Assessment

**DOI:** 10.1007/s12011-026-05055-z

**Published:** 2026-03-19

**Authors:** Mehmet Emin Şeker, Ayşegül Erdoğan, Duygu Nur Çobanoğlu, İlginç Kızılpınar Temizer

**Affiliations:** 1 School of Tobacco Expertise, Manisa Celal Bayar University, Manisa, Türkiye; 2 Application and Research Centre for Testing and Analysis (Ege MATAL), Ege University, İzmir, Türkiye; 3 Vocational School of Food, Agriculture and Livestock, Bingöl University, Bingöl, Türkiye; 4 Vocational School of Health Services, Giresun University, Giresun, Türkiye

**Keywords:** Monofloral bee pollen, melissopalynological analysis, elemental composition, health risk assessment, XPS, ICP-MS

## Abstract

Monofloral bee pollen, collected by honeybees from a single dominant botanical source, enables a precise and reproducible evaluation of elemental composition due to high taxonomic purity and reduced matrix variability. This study aimed to (i) elucidate the relationships between botanical origin, physicochemical properties, and mineral and PTE accumulation in monofloral pollen samples (MP1–MP10) collected from Bingöl, Türkiye, and (ii) the non-carcinogenic and carcinogenic health risks for adults and children under realistic daily intake scenarios. Melissopalynological analysis confirmed high monoflorality (≥ 98%). ICP-MS results showed significant inter-species differences (*p* < 0.05), particularly for Mg, Fe, and Mn, indicating strong botanical and geochemical control over mineral uptake. XPS profiling verified a conserved C/O-rich exine structure while supporting surface-level chemical fingerprinting across pollen types. Target Hazard Quotient (THQ) and cumulative Hazard Index (HI) values confirmed that all adult and child exposure cases remained within safe limits (HI < 1). Carcinogenic risk estimates for Cd, Ni, Pb, and As were below acceptable thresholds for adults (CR < 1 × 10^− 4^), whereas Cr exceeded the standards in certain child groups (CR > 1 × 10^− 4^), reflecting age-dependent sensitivity despite trace-level occurrence. Several monofloral pollens supplied nutritionally relevant Fe and Mn contributions toward RDA (%), reinforcing their dietary value.

## Introduction

The close relationship between honeybees and flowering plants is a well-known example of mutualism-driven co-evolution [1]. Honeybees (*Apis mellifera*) are recognized as generalist foragers, collecting nectar and pollen from many botanical sources. However, the dominant forage taxa may vary depending on geography, season, and resource availability, which makes botanical-source characterization essential for understanding pollen origin and ecosystem interactions [2]. The families *Asteraceae*,* Rosaceae*,* Dipsacaceae*,* and Fabaceae* are repeatedly highlighted as important nectar and pollen reservoirs, supporting seasonal foraging continuity and ecological balance [3, 4].

Pollen grains, the male gametophytes of plants, possess distinctive morphology and surface ornamentation, which influence their interaction with the environment [5]. The pollen surface is protected by the exine layer, composed mainly of sporopollenin, a chemically resilient and inert biopolymer [6]. Structural ornamentations such as spines, granules, striations, and reticulations expand surface contact area, supporting adhesion to the bee body and interaction with soil-air inputs. Beyond morphology, elemental accumulation in pollen is strongly affected by plant physiology, soil geochemistry, atmospheric deposition, and seasonal environmental stressors [7]. After being harvested by honeybees, biochemical exposure to nectar blending, enzymatic activity, and hive storage may further modify measurable mineral loads. Because of this dual influence, pollen carries both nutritional minerals (e.g., Mg, Ca, Fe, Mn, Zn) and trace-level environmental inputs, including potentially toxic elements (Pb, Cd, Cr, Ni, and As, ) making long-term exposure evaluation necessary, particularly for children [8–15]. Although many studies focus on bulk elemental composition, investigations combining surface-sensitive analysis with long-term health-risk metrics for authenticated monofloral pollen remain scarce. Surface techniques alone cannot quantify total mineral load, while bulk methods cannot provide exine-level resolution [16–21]. Therefore, this study employed a surface-to-bulk analytical approach, integrating XPS and ICP-MS to reduce analytical uncertainty and enhance elemental comparisons based on botanical sources. The oxygen-containing functional groups on the pollen exine may also facilitate the surface retention and adsorption of elements that result from air pollution, hence elucidating species-specific elemental variations, especially at trace levels [20]. X-ray Photoelectron Spectroscopy (XPS) was utilized to characterize the outer exine surface of monofloral pollen, whereas Inductively Coupled Plasma Mass Spectrometry (ICP-MS) was employed to measure total dry-weight mineral and potentially toxic element (PTE) contents (mg kg⁻¹) This multi-technique methodology enhances the reliability of elemental fingerprinting and enables life-stage-aware exposure interpretation compared to single-layer analysis alone.

Monofloral pollen, verified at a purity level of 98% or higher, offers a more consistent basis for elemental analysis due to less matrix variability and reduced botanical interference [22, 23]. In contrast, polyfloral pollen, due to its mixed taxonomic structure, may obscure source-dependent mineral trends. For this reason, monofloral pollen enables more standardized nutritional and safety evaluation across life stages. In the present study, ten monofloral bee pollen varieties (MP1–MP10) collected from Bingöl-Türkiye were analyzed to (i) determine species-dependent elemental variability, (ii) quantify nutritional contribution via RDA (%), and (iii) evaluate non-carcinogenic (THQ, HI) and carcinogenic (CR) exposure safety under realistic chronic dietary intake assumptions. This integrated evaluation highlights that monofloral bee pollen offers nutritionally valuable minerals, but long-term safety interpretation should consider life-stage susceptibility, particularly in children, even when contaminants are present at trace levels.

## Materials and Methods

### Collection of Bee Pollen Samples and Palynological Analyses

The bee pollen sample was collected during the beekeeping season between May and August 2022 from a special apiary located in the Solhan district (N 39° 01.996’ E 040° 53.428’) of Bingöl. Solhan district is situated in the eastern part of Bingöl in Eastern Anatolia. Phytogeographically, the area falls within the Iran-Turan floristic region, characterized by deciduous forests and steppe vegetation. The district has an average of 1395 m, while the surrounding mountainous areas. The climate is characterized by cold and snowy winters and warm to hot, relatively dry summers.

The samples were collected from ten different hives of the same honeybee race within the same apiary, with a distance of 7 m between hives. The samples were transported to the laboratory under cold chain conditions. First, they have been manually sorted by color, and pollen pellets of the same color have been grouped and combined (approximately 10 g per sample). And then, the slides were prepared according to the method described by Cobanoglu [24], Çobanoğlu, Kizilpinar Temizer [25]. These samples were examined under a light microscope (Leica 2500 DM) to assess their botanical origins and purity. Purity refers to designating the same plant taxon to which the pollen grains in each sample, per g, belong. For bee pollen classified as monofloral, a purity value greater than 98% was assumed acceptable [23, 26, 27]. The terminology employed by Punt, Hoen [28] was utilized for description of pollen morphology. The morphology of pollen grains in this study belonging to the identified taxa was investigated by light microscopy and further characterized using scanning electron microscopy (SEM). Also, plant samples that are foraged by bees were collected for palynological identification across the beehives. Also, plant samples that are foraged by bees were collected for palynological identification across the beehives [29].

### Surface Elemental Composition of MP sampless by X-ray Photoelectron Spectroscopy (XPS)

XPS measurements were performed using Thermo Scientific K-Alpha XPS instrument equipped with monochromatic Al Kα radiation (1486.7 eV). The binding-energy scale was calibrated to the C 1s peak at 284.8 eV. Survey scans were acquired at a pass energy of 200 eV with a 1.0 eV step size (0–1350 eV range). The X-ray spot size was fixed at 300 μm for single-point analysis, and three independent surface points per sample were analyzed to ensure spatial representativeness. Peak deconvolution, curve fitting, data processing, and quantification were performed using Avantage Software (v5.9915). The base pressure in the analysis chamber was maintained at 5 × 10⁻⁹ mbar. High-resolution spectra of all detected elements were evaluated to assign surface chemical states, with C 1s spectra deconvoluted to resolve exine-associated bonding environments. The full spectral dataset was interpreted based on both survey and high-resolution scans for all identified surface elements.

### Preparation of MP Samples for ICP-MS Measurements

In order to avoid the moisture absorption of monofloral pollen samples, they were kept in glass bottles and stored at − 20 °C. Finally, they were dried at 40 °C in the oven until constantly weighed and grounded with the aid of a mortar prior to acid digestion. Then, they were subjected to closed-vessel high-pressure microwave-assisted digestion to achieve complete mineralization of their elemental content according to the methods applied by Şeker, Erdoğan [30]. To decompose the organic matrix of dried monofloral bee pollens and quantitatively release the embedded elements into solution, closed-vessel, high-pressure microwave-assisted wet digestion (MWD) was employed. Accurately weighed pollen samples (0.25 g) were placed in PFA (perfluoroalkoxy) Teflon digestion vessels, followed by the addition of 2.0 mL of a freshly prepared HNO₃/HCl acid mixture (5:2, v/v) [4, 31]. Microwave digestion was performed using a MARS 5 system (CEM Corporation, USA) at 1600 W and 210 °C for 20 min. After digestion, vessels were allowed to cool to room temperature. The digests were homogenized by vortex mixing and diluted with ultrapure deionized water to a final volume of 10 mL prior to ICP-MS analysis. The analytical method applied in this study was previously validated using certified reference material (BCR^®^279, sea lettuce), and its accuracy, precision, recovery, and detection limits were comprehensively reported by Şeker, Erdoğan [30]. All digestion experiments were conducted in triplicate (*n* = 3), and analytical precision was evaluated by calculating the relative standard deviation (RSD, %) for each pollen sample.

### ICP-MS Analysis of Elements

A total of 13 elements, encompassing both essential minerals and potentially toxic elements (Mn, Fe, Zn, Cu, Na, Mg, Ca, Se, Cr, Pb, Cd, As and Ni), were identified in the analyzed monofloral pollen samples using an inductively coupled plasma mass spectrometry (ICP-MS) instrument (Bruker 820-MS ICP-MS spectrometer) according to the procedure validated previously Şeker, Erdoğan [30]. Internal standards including ⁴⁵Sc, ⁸⁹Y, ¹⁰³Rh, ¹¹⁵In, and ²⁰⁹Bi were employed to correct for matrix interferences and instrumental drift, thereby ensuring high analytical accuracy and precision. The ICP-MS operating parameters were optimized as follows: ICP-MS plasma conditions were given as follows: plasma flow: 16.50 L min^− 1^, auxiliary flow: 1.65 L min^− 1^, sheath gas flow: 0.20 L min^− 1^, nebulizer flow: 0.98 L min^− 1^, and sampling depth: 6.00 mm, power: 1.40 kW. Prior to sample analysis, multi-point calibration curves were constructed for each element. Eight calibration levels (0.001, 0.002, 0.005, 0.1, 0.2, 0.5, and 1.0 mg L⁻¹) were prepared from certified standard solutions to establish linearity and sensitivity. Each measurement was performed in quadruplicate, and the mean values were calculated to minimize experimental errors. The final elemental concentrations in the samples were obtained by applying the dilution factors to the values derived from the calibration curves. This procedure ensured reliable quantification of both macro- and micro-elements, as well as potentially toxic elements, providing a comprehensive profile of the elemental composition in the samples.

### Statistical analysis

Statistical analyses of the sample variables were carried out using different methodological approaches. Descriptive statistics were expressed as mean ± standard deviation, and general statistical evaluation was performed with SPSS (version 26.0, USA). Multivariate analyses—including heatmap and Principal Component Analysis (PCA)—were conducted in (version 4.3.x; R Core Team, 2023) RStudio (Posit Software, PBC). Heatmap visualization employed pheatmap, vegan, and RColorBrewer, while PCA was performed with factoextra and FactoMineR.

### Recommended Mineral Intake for Female and Male

There is no specified or recommended daily intake of bee pollen; however, consumption levels are frequently compared to dietary reference intakes for macronutrients and micronutrients. Numerous studies suggest for a moderate daily consumption of 20 to 40 g for adults to yield advantageous nutritional benefits while being under acceptable thresholds for potentially harmful substances [32–35]. Consequently, assessing bee pollen intake in relation to the Recommended Dietary Allowance (RDA) is essential for optimizing nutritional advantages and minimizing health hazards linked to contaminant exposure. This work employed a conversion factor (Cf:0.085) to quantify the contributions of pollen minerals to %RDA values, limiting the increase attributed to drying [36]. The conversion factor employed in toxicity calculations was similarly utilized to determine the contribution to %RDA values. Consequently, the values provided correspond to fresh pollen.

### Health Risk Assessment

Bee pollen is a nutritionally rich food source, containing essential macro and micronutrients [3, 11]. However, it can also accumulate potentially toxic elements (PTEs), raising concerns about potential health risks [14, 37]. Several studies have assessed the elemental composition of bee pollen samples from different regions, finding varying levels of essential and non-essential elements [9, 18, 36]. Risk assessments using metrics such as estimated daily intake (EDI), target hazard quotient (THQ), hazard index (HI), and target cancer risk (CR) generally indicate that consuming recommended amounts of bee pollen poses minimal non-carcinogenic risks for adults and children [3, 11]. However, some studies have identified potential carcinogenic risks associated with certain elements, particularly for children [9, 14]. Bee pollen is also recognized as a valuable bioindicator for environmental contamination [38].

The ‘tolerable daily intake’ (TDI) refers to the amount of a substance present in food, water, air, or beverages that can be ingested throughout a lifetime without posing appreciable health risks, as defined by the European Food Safety Authority [39–41]. In this study, the estimated daily intake (EDI) of metals from bee pollen was determined using reported data on average pollen consumption and dietary patterns, in line with previous studies on bee product intake [41–43]. The EDI, expressed in milligrams per kilogram of body weight per day, was calculated according to Formula 1:

$$EDI=\frac{IRd\times\mathrm{MC}}{BW}\times10^{-3}1$$

IRd represents the daily average consumption of bee pollen (40 mg/day for adult and 20 mg/day for child), MC indicates the metal concentration in bee pollen (mg/kg), and BW denotes the average adult body weight (70 kg for adult and 15 kg for child) [9, 44]. The target hazard quotient (THQ) was applied to evaluate the non-carcinogenic health risks associated with specific metal pollutants. THQ is defined as the ratio between the estimated daily intake (EDI) of a pollutant and its corresponding reference dose (RfD) established by regulatory authorities. A THQ value greater than one indicates a potential risk of adverse health effects due to exposure. Accordingly, the THQ for metals in bee pollen was calculated to assess non-carcinogenic risks and to highlight possible health concerns linked to the regular consumption of contaminated pollen. The calculation was performed using Formula 2:

$$THQ=\frac{EF\times\mathrm{ED}\times\mathrm{IRd}\times\mathrm{MC}}{RfD\times\mathrm{BW}\times{AT}_{noncancer}}\times10^{-3}\times\mathrm{Cf}2$$

In this context, EF represents the exposure frequency, generally set at 350 days per year, while ED denotes the exposure duration, commonly established as 26 years for non-carcinogenic risk assessments, following the guidelines of the United States Environmental Protection Agency [45]. BW is the average adult body weight (70 kg), child body weight (15 kg), and AT corresponds to the average exposure time for non-carcinogenic substances, calculated as 365 days/year × 26 years. RfD refers to the oral reference dose, which is used to evaluate the potential health risks associated with a given substance. To estimate the overall non-carcinogenic risk from simultaneous exposure to multiple metals, the hazard index (HI) was calculated by summing the THQ values of all detected pollutants in bee pollen samples. An HI value greater than one suggests a potential health risk due to the cumulative effects of these pollutants. The calculation of HI was performed using Formula 3:

$$HI={\sum}_{i=1}^{n}{THQ}_{i}3$$

Target cancer risk (CR) is a key parameter used to estimate the probability of carcinogenic effects arising from exposure, and it provides essential guidance for risk management strategies and regulatory decision-making. The calculation of CR incorporates factors such as exposure frequency and duration, carcinogen potency, and individual susceptibility, offering a more refined assessment of potential cancer risks associated with environmental contaminants. This comprehensive evaluation facilitates informed public health decisions aimed at minimizing long-term hazards. In this study, carcinogenic risks were quantified through the CR metric, which was calculated using Formula 4:

$$CR=\frac{EF\times\mathrm{ED}\times\mathrm{IRd}\times\mathrm{MC}\times\mathrm{CSF}}{BW\times{AT}_{cancer}}\times10^{-3}\times\mathrm{Cf}4$$

The carcinogenic slope factor (CSF) values for oral exposure, expressed in (mg kg⁻¹ day⁻¹), were obtained from the Integrated Risk Information System (IRIS) and are as follows: Pb = 0.0085, Cd = 0.38, Ni = 1.7, and Cr = 0.5 [46–48].

## Results and Discussion

### Palynological Evaluation

**Fig. 1:**
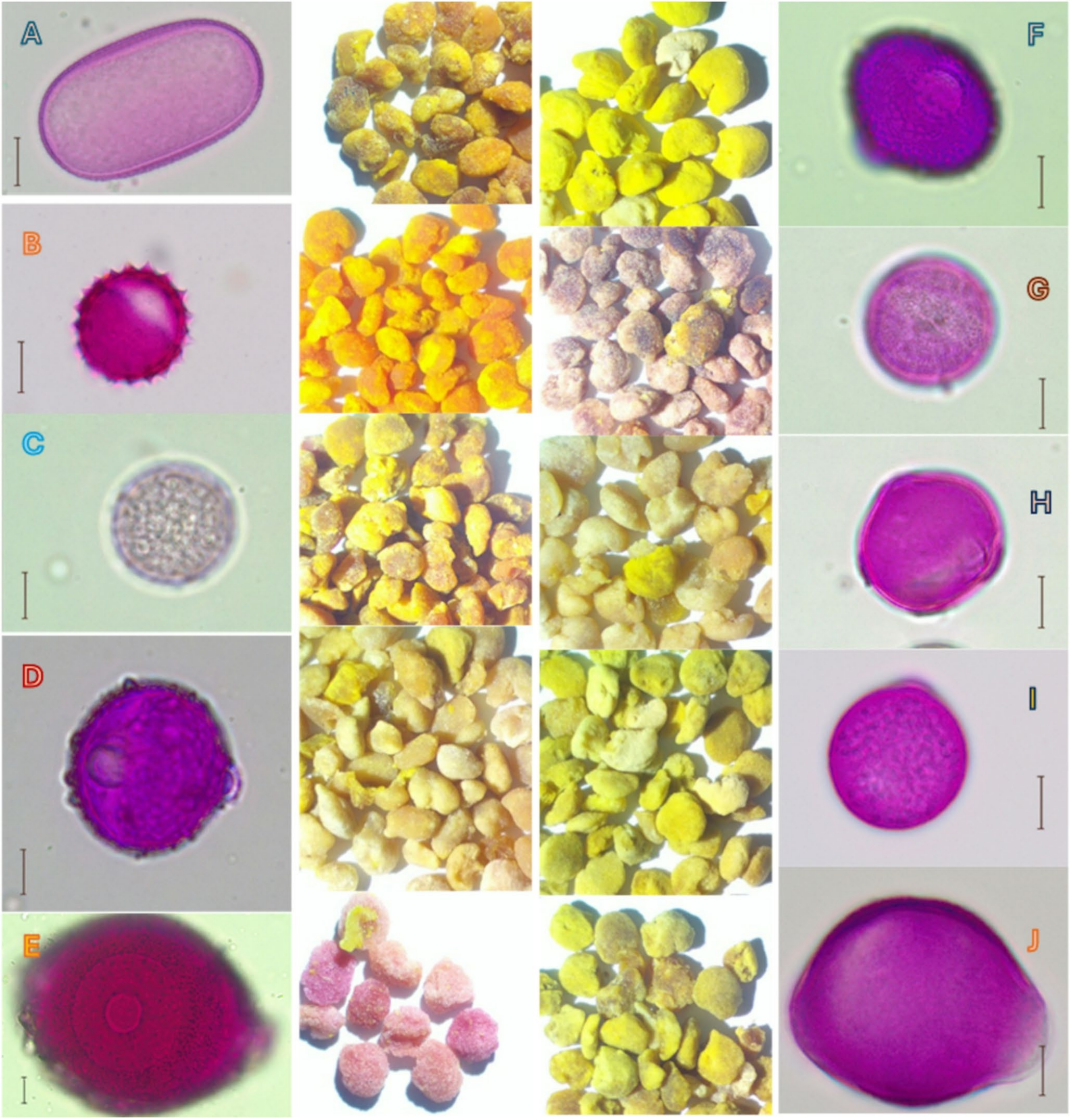
Stereo microscope photograph of pollen pellets and Light microscopic photograph of monofloral pollen grains. **A** Acanthus sp.; **B** Helicrysum sp.; **C** Plantago sp.; **D** Centaurea sp.; **E** Cephalaria sp., **F** Trifolium sp., **G** Papaver sp.; **H** Astragalus sp.; **I** Sanguisorba sp.; **J** Rosa sp.

**Fig. 2:**
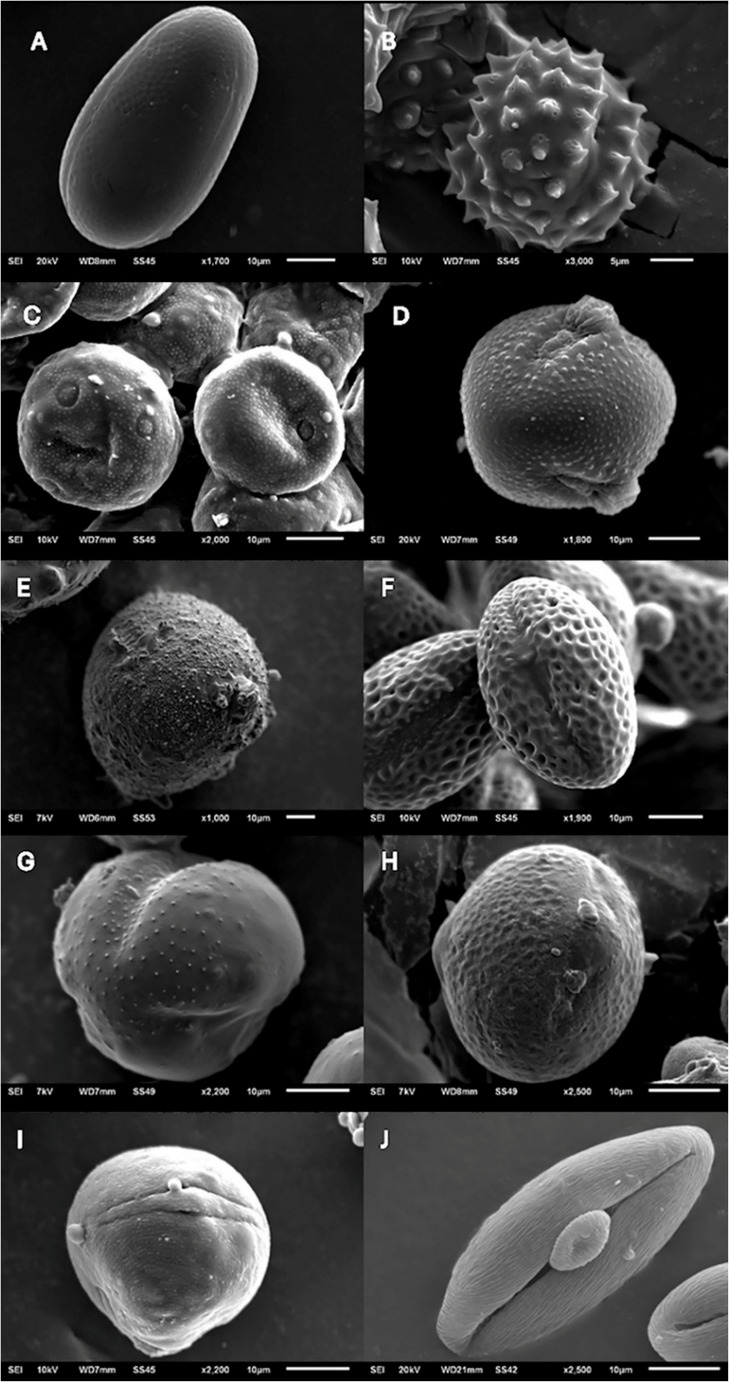
Scanning Electron Microscope (SEM) images of monofloral pollen grains. **A** Acanthus sp.; **B** Helicrysum sp.; **C** Plantago sp.; **D** Centaurea sp.; **E** Cephalaria sp., **F** Trifolium sp., **G** Papaver sp.; **H** Astragalus sp.; **I** Sanguisorba sp.; **J** Rosa sp.

Following methodological approach of Campos, Anjos [49], bee pollen samples were separated according to their color (Fig. 1). According to palynological examination of pollen grains, ten melliferous taxa belonging to seven families were observed under light microscope. Morphological data of BP samples are summarized in Table 1. The results of this study indicated that honeybees exhibit preference to visit for foraging *Acanthaceae*,* Asteraceae*,* Caprifoliaceae*, *Dipsicaceae*,* Fabaceae*,* Papaveraceae*, and *Rosaceae* families (Fig. 2). *Asteraceae* (two taxon), and *Fabaceae* (two taxon) were highly visited by honeybee as foraged source as like the findings by Çobanoğlu, Kizilpinar Temizer [25] The pollen morphology is used for determining the melliferous plants, and explaining the honeybees forage behaviours [50]. In the assessed samples, most of the dominant aperture types were tricolporate and tricolpate as previously identified by Temizer [3]. According to Pernal [51], bees preferred particle sizes below 150 μm, showing the strongest response to those smaller than 45 μm; in our study, pollen size also appeared to play a significant role, as medium-sized grains were detected. Furthermore, ornamentation patterns of pollen grains affected the foraging preferences of honeybees. Echinate and microechinate exine surfaces, characteristics of Asteraceae (*Helicrysum*,* Centaurea*), *Caprifoliaceae* (*Cephalaria*), and *Papaveraceae* (*Papaver*) were highly represented, possibly because their spins improve adhesion to the bee’s body. As previously studied by Reis, Santos [52], the exine ornamentation mostly exhibited ranging from striate to echinate. However, it is important to note that pollen morphology interacts with ecological and behavioral factors such as floral abundance, phenology, and nectar rewards.

**Table 1:** Palynological analysis results according to identification of pollen grains, monoflorality, and morphological analyses

Sample	Pollen Taxa/Monoflorality (%)	Family	Aperture	Polar axis (*P*)	Equatorial axis	Pollen shape /(*P*/E)	Ornemantation
MP01	*Acanthus* sp./ 98.3	Acanthaceae	Tricolpate	54.95 ± 2.58	31.04 ± 2.74	Prolate/(1.8)	Reticulate
MP02	*Helicrysum* sp./99.2	Asteraceae	Tricolporate	22.97 ± 5.21	20.63 ± 3.12	Prolate spheroidal (1.11)	Echinate
MP3	*Plantago* sp. / 99.4	Plantaginaceae	Pantoporate	28.64 ± 2.15	28.20 ± 2.08	Spheroidal (1.01)	Verrucate
MP4	*Centaurea* sp./99.3	Asteraceae	Tricolporate	39.10 ± 4.13	35.84 ± 3.50	Prolate spheroidal (1.09)	Scabrate
MP05	*Cephalaria* sp. /99.8	Caprifoliaceae	Triporate	60.80 ± 7.80	73.54 ± 10.51	Suboblate (0.8)	Echinate, microechinate
MP06	*Trifolium* sp./98.9	Fabaceae	Tricolporate	41.53 ± 2.30	27.09 ± 3.40	Prolate (1.53)	Reticulate perforate
BP07	*Papaver* sp./98.9	Papaveraceae	Tricolpate	28.01 ± 3.22	30.79 ± 4.03	Spheroidal (0.9)	microechinate, perforate
MP08	*Astragalus* sp. /99.1	Fabaceae	Tricolporate	30.70 ± 3.70	27.45 ± 3.81	Prolate spheroidal (1.1)	Reticulate
MP09	*Sanguisorba* sp. /99.0	Rosaceae	Tricolporate	31.02 ± 2.80	32.55 ± 3.00	Spheroidal (0.95)	Striate, microechinate
MP10	*Rosa* sp./99.5	Rosaceae	Tricolporate	33.43 ± 3.40	27.30 ± 2.50	Subspheroidal	Striate, perforate

### Surface Chemistry of MP samples

**Fig. 3:**
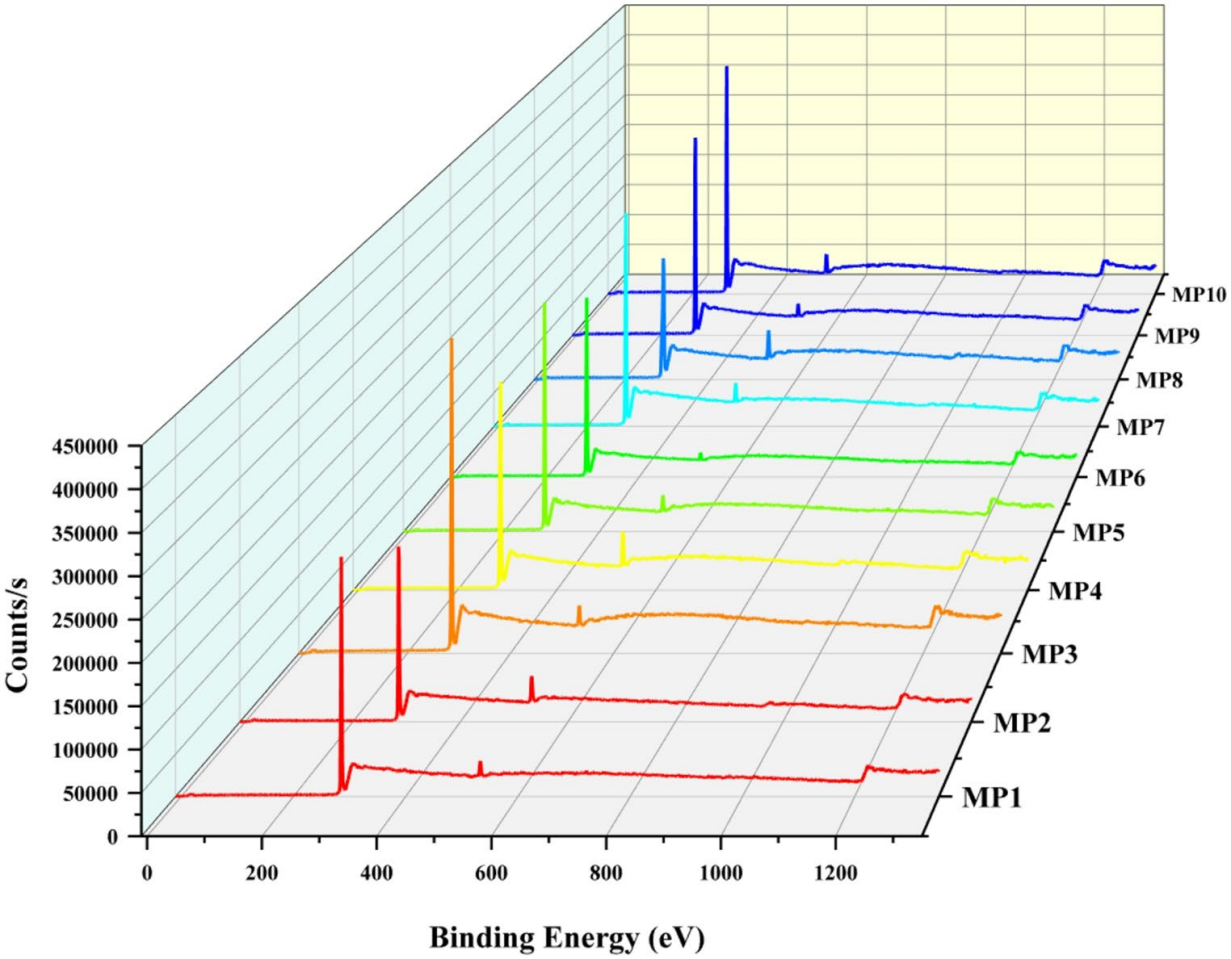
XPS survey spectra for monofloral pollen samples

The XPS survey spectra of monofloral bee pollen samples were visualized as a 3D stacked plot (Fig. 3) to comparatively profile the elemental composition of the outer exine layer across ten distinct pollen sources (MP1–MP10). The survey scans (0–1300 eV, Counts/s) reveal strong photoelectron responses overwhelmingly dominated by carbon and oxygen core-level emissions, consistent with the conserved sporopollenin-based organic architecture of the pollen exine matrix. X-ray photoelectron spectroscopy (XPS) peaks at ~ 285 eV (C 1s) and ~ 530–532 eV (O 1s) definitively confirm the complex surface chemistry of sporopollenin, revealing oxygenated functional groups and surface-adhered biomolecules. The evidence is robust: Mackenzie [53] confirms sporopollenin’s surface possesses diverse functional groups including phenolic, alkane, ketone, and carboxylic acid groups. Ahlers, Bubert [54] specifically noted that oxygen in sporopollenin originates primarily from hydroxyl groups in aliphatic structures. Mikhael, Jurcic [55] further substantiates the complex surface chemistry, showing a poly(hydroxyacid) network with multiple functional groups. The XPS peaks precisely indicates these oxygen-containing functional groups (C–O, C = O, O–C = O), while also detecting minor biomolecular contributions like lipids, proteins, and saccharides (C 1s spectra not shown). Survey-level quantification (Table 2) further supports this spectral observation, showing carbon as the dominant surface element (93–98.5 at%) and oxygen at lower but consistent levels (1.5–6.99 at%), indicative of subtle inter-species variability in surface oxidation, functional group density, or adsorption of environmental polar moieties. Notably, MP6 exhibited the highest C% (98.5%) with the lowest O% (1.5%), suggesting a comparatively more hydrophobic and less oxidized exine surface, whereas MP2, MP4, MP8, and MP5 displayed the highest oxygen proportions (~ 7%), implying relatively greater surface functionalization or adsorption of polar biomolecules. Although minor spectral spikes were intermittently observed—attributable to charging effects inherent to highly insulating biological samples—energy referencing to the adventitious carbon peak at 284.8 eV ensured reliable alignment of binding energies. The low standard deviation values (*n* = 3 independent point measurements per sample) indicate good surface homogeneity and measurement repeatability; however, near-saturation of carbon in some samples may obscure trace heteroatoms below survey detection limits. Collectively, the 3D spectral layout and atomic % surface data validate that each monofloral pollen exhibits a distinct surface chemical fingerprint driven primarily by variations in oxygenated functionalization and charging behavior, while maintaining a conserved organic exine framework dominated by C and O.

**Table 2:** Quantitative composition (atomic %) of monofloral pollen sample surfaces determined by XPS

MP samples	C %	O%
MP1	96.06 ± 0.57	3.94 ± 0.22
MP2	93.03 ± 0.46	6.97 ± 0.34
MP3	96.51 ± 0.68	3.49 ± 0.40
MP4	93.01 ± 0.49	6.99 ± 0.53
MP5	96.15 ± 0.38	3.85 ± 0.17
MP6	98.50 ± 0.52	1.50 ± 0.21
MP7	96.25 ± 0.64	3.75 ± 0.43
MP8	93.20 ± 0.21	6.80 ± 0.67
MP9	97.58 ± 0.91	2.42 ± 0.57
MP10	95.88 ± 0.88	4.12 ± 0.28

The number of replicates was 3

The standard deviations are given as ± values

From the combined evaluation of palynological data and XPS surface analysis, it is evident that monofloral pollen samples exhibit clear taxon-specific morphological differentiation, whereas their surface chemistry remains largely conserved. XPS results show that all pollen types share a sporopollenin-dominated organic exine with only minor interspecific variation, indicating that surface chemical composition does not mirror palynological differentiation. Thus, XPS confirms the presence of a common surface chemical framework across different floral sources, while taxon-specificity is primarily expressed at the morphological level.

Djubina, Jansone [56] used scanning electron microscopy to detect surface chemical elements (O, C, K, P, Ca, Pb, Zn, Sn) on pollen walls and reported pollutant elements such as Pb, Zn, and Sn on pollen surfaces, suggesting that pollen grains can act as carriers of environmental contaminants. However, the available sources provide limited quantitative data on the strength of correlation between surface chemistry properties and elemental distribution patterns.

In a related XPS-based study, the chemical nature of pollen surfaces was further resolved by identifying oxygen-containing functional groups on the exine, and Pb retention was explained through interactions with these surface functional moieties [30]. Similarly, in the present study, XPS analysis revealed a sporopollenin-dominated pollen surface enriched with oxygenated functional groups, confirming that surface chemistry is primarily defined by functional group composition rather than by inorganic phases. Together, these findings support the interpretation that pollutant elements detected on pollen surfaces may be associated with interactions involving surface functional groups, even when their concentrations are not sufficient to form a dominant surface chemical phase.

In the reported SEM-EDS study above [56], inorganic elements on pollen walls were detected; however, the micrometer-scale penetration depth of EDS inherently integrates signals from surface, subsurface, and near-bulk regions. In contrast, the nanometer-scale surface sensitivity of XPS employed in the present study specifically probes the outermost exine layer. The dominance of carbon and oxygen signals in XPS survey spectra therefore indicates that, although elements may be present within or beneath the pollen wall as detected by SEM–EDS, they do not constitute a dominant chemical phase at the true pollen surface. This scale-dependent distinction helps explain the limited correlation between surface chemistry and elemental distribution reported in the literature and supports the interpretation that elemental enrichment in pollen is primarily internal rather than surface-driven.

### Element Profile

In this study, the elemental composition of ten monofloral bee pollen samples (MP1–MP10) was expressed as mean ± standard deviation (mg kg⁻¹) based on three independent analytical replicates, and inter-sample variability was evaluated using one-way ANOVA followed by Tukey’s post-hoc test (*p* < 0.05) (Table 3). The results reveal substantial botanical and environmental heterogeneity in both macro- and micro-element concentrations, with statistically significant differences detected for nearly all quantified elements (*p* < 0.05). Among macro-elements, Mg exhibited the widest concentration span (585–2970 mg kg⁻¹), followed by Na (25.6–97.6 mg kg⁻¹) and Ca (89–262 mg kg⁻¹), indicating that pollen mineral accumulation is strongly source-dependent and influenced by plant physiology, soil geochemistry, and environmental exposure. In the micro-element category, Fe (115–546 mg kg⁻¹) and Mn (16–112 mg kg⁻¹) showed particularly strong differentiation among pollen types, supporting their potential role as discriminative chemical markers in pollen source profiling. Essential nutritional elements such as Zn, Cu, Se, and Mn also varied significantly, reflecting species-specific micronutrient uptake dynamics. Potentially toxic elements (Pb, Cd, As, Ni, Cr) were detected mostly at low or ultra-trace levels, yet still displayed statistically distinct profiles between pollen sources, demonstrating that low abundance does not equate to compositional uniformity, and that PTE distributions remain source-specific within the analyzed sample set.

**Table 3:** Content (mg kg^−1^DW) of microelements, macroelements, and PTEs determined in monofloral pollen samples

Samples	Na	Mg	Ca	Mn	Fe	Zn	Cu	Se	Ni	Cr	Pb	Cd	As
MP1	26.09 ± 0.58^h^	2286.90 ± 34.24^b^	89.00 ± 3.61^g^	62.35 ± 2.66^b^	545.99 ± 5.59^a^	87.49 ± 0.97^a^	23.61 ± 0.04^a^	0.04 ± 0.00^e^	< 0.0001	6.00 ± 0.18^b^	< 0.0001	0.36 ± 0.02^a^	0.05 ± 0.00^b^
MP2	44.02 ± 0.07^e^	585.36 ± 18.74^h^	152.00 ± 3.61^e^	24.38 ± 1.33^e^	218.06 ± 3.92^f^	41.90 ± 0.59^h^	11.38 ± 0.02^h^	0.02 ± 0.00^h^	< 0.0001	0.65 ± 0.03^d^	< 0.0001	0.15 ± 0.02^b^	0.02 ± 0.00^fg^
MP3	25.60 ± 0.03^h^	1322.08 ± 14.39^d^	113.67 ± 4.16^f^	51.65 ± 2.28^c^	456.50 ± 8.70^b^	62.80 ± 0.47^b^	18.92 ± 0.03^c^	0.03 ± 0.00^f^	< 0.0001	5.54 ± 0.40^b^	< 0.0001	0.37 ± 0.03^a^	0.05 ± 0.00^b^
MP4	97.62 ± 0.32^a^	985.13 ± 5.80^f^	262.00 ± 11.36^a^	34.76 ± 1.78^d^	206.12 ± 3.24^f^	58.95 ± 0.38^d^	21.05 ± 0.05^b^	0.05 ± 0.00^d^	< 0.0001	2.89 ± 0.04^c^	0.41 ± 0.01	0.17 ± 0.01^b^	0.04 ± 0.00^c^
MP5	37.64 ± 0.16^f^	2969.96 ± 17.01^a^	107.67 ± 2.52^f^	16.30 ± 1.47^f^	115.29 ± 2.00^h^	53.70 ± 0.54^e^	16.10 ± 0.05e	0.05 ± 0.00^b^	< 0.0001	< 0.0001	< 0.0001	0.04 ± 0.00^c^	0.02 ± 0.00^g^
MP6	54.40 ± 0.66^d^	1778.00 ± 14.11^c^	256.67 ± 5.86^a^	34.59 ± 1.23^d^	255.47 ± 3.53^e^	48.20 ± 0.42^g^	12.06 ± 0.07^gh^	0.07 ± 0.00^a^	< 0.0001	2.26 ± 0.08^c^	< 0.0001	0.36 ± 0.00^a^	0.02 ± 0.00^ef^
MP7	68.66 ± 0.35^c^	1002.00 ± 13.75^f^	105.67 ± 4.04^f^	51.19 ± 2.01^c^	358.23 ± 6.83^c^	51.02 ± 0.22^f^	17.12 ± 0.07^d^	0.07 ± 0.00^a^	< 0.0001	< 0.0001	< 0.0001	0.17 ± 0.01^b^	0.06 ± 0.00^a^
MP8	77.41 ± 0.25^b^	861.00 ± 9.17^g^	232.00 ± 3.61^b^	28.39 ± 1.77^e^	185.79 ± 3.41^g^	49.04 ± 0.18^g^	18.78 ± 0.05^c^	0.05 ± 0.00^c^	< 0.0001	2.54 ± 0.07^c^	< 0.0001	0.15 ± 0.00^b^	0.04 ± 0.00^d^
MP9	29.47 ± 0.47^g^	1234.67 ± 15.57^e^	184.67 ± 5.69^d^	112.27 ± 1.96^a^	339.87 ± 4.96^d^	54.54 ± 0.07^e^	14.28 ± 0.02^f^	0.02 ± 0.00^g^	< 0.0001	10.88 ± 0.65^a^	< 0.0001	0.14 ± 0.00^b^	0.02 ± 0.00^f^
MP10	39.06 ± 0.05^f^	1308.67 ± 27.30^d^	216.00 ± 5.57^c^	113.95 ± 2.58^a^	259.06 ± 5.02^e^	60.87 ± 0.52^c^	12.42 ± 0.03^g^	0.03 ± 0.00^f^	< 0.0001	< 0.0001	< 0.0001	0.05 ± 0.00^c^	0.03 ± 0.00^e^
Total	50.00 ± 4.27	1433.38 ± 698.17	171.93 ± 64.65	52.98 ± 33.41	294.04 ± 127.09	56.85 ± 12.06	16.57 ± 0.04	0.04 ± 0.02	< 0.0001	3.08 ± 3.39	< 0.0001	0.20 ± 0.12	0.03 ± 0.01

Different superscript letters within the same column indicate significant differences between means according to ANOVA followed by Tukey’s multiple comparison test (*p* < 0.05)

Monofloral bee pollen samples exhibit distinct elemental compositions dominated by macroelements, with specific profiles varying by botanical origin. Several studies to analyze monofloral bee pollen elemental composition. Monofloral bee pollen samples exhibit distinct elemental compositions dominated by macroelements, with specific profiles varying by botanical origin. N. Lilek et al., 2021 found that monofloral samples (sweet chestnut, maple, dandelion, rapeseed, flowering ash, buckwheat, common ivy, and plantain) contained P, S, Cl, K, Ca, Mn, Fe, Zn, Br, Rb, and Sr, with K, P, S, Ca, and Cl being most abundant, followed by Fe, Mn, Zn, Rb, Br, and Sr. El Ghouizi Asmae et al., 2021 identified potassium and magnesium as the most abundant minerals in eight monofloral samples from Morocco. Çobanoğlu, Kizilpinar Temizer [25] analyzed 31 elements in monofloral Verbascum samples using ICP-MS, while A. Kostić et al., 2022 quantified 17 micro/trace elements in monofloral sunflower pollen via ICP-OES. Temizer [22] reported K, P, Ca, Mg, and Na as the most abundant macroelements across different floral sources, with Ti, Ba, Ni, Cr, and V being the least abundant ultratrace elements. Lilek, Kandolf Borovšak [57] found statistically significant differences in elemental content between monofloral types according to botanical origin, which they attributed to differences in soil and plant elemental composition and plant metabolism. This indicates that each monofloral type has a characteristic elemental fingerprint reflecting its specific botanical source. Asmae, Nawal [58] reported that heavy metals were generally not detected in monofloral samples, except for very small amounts of lead in two samples, while Kostić, Dojčinović [59] found some potentially toxic elements in monofloral sunflower pollen requiring health risk assessment.

The overall C/O surface dominance noted in survey scans (Table 2) explains the insulating nature of pollen exine but was not the focus of bulk interpretation. Collectively, the findings confirm that monofloral pollen types possess significantly different elemental profiles shaped by botanical origin and environmental conditions, reinforcing the importance of source-based evaluation in nutritional quality and PTE risk comparisons.

Overall, the XPS and ICP-MS results indicate that surface chemistry and elemental composition of monofloral bee pollens reflect different but biologically coordinated levels of pollen organization. XPS demonstrates that all samples share a conserved, carbon-dominated sporopollenin exine with limited yet taxon-dependent variation in oxygenated functional groups, confirming that the pollen surface is largely organic and does not host abundant inorganic phases. In contrast, ICP-MS reveals pronounced and statistically significant differences in macro-, micro-, and trace element concentrations among pollen types, indicating that elemental fingerprints are primarily governed by internal biological accumulation mechanisms and source-specific plant–environment interactions. The absence of strong inorganic signals in XPS, despite clear elemental differentiation in ICP-MS, suggests that any surface-associated elements occur at low concentrations below XPS survey detectability, and that bulk elemental profiles are not controlled by surface enrichment processes. Thus, the combined use of XPS and ICP-MS provides complementary, multi-scale evidence that both surface chemical architecture and elemental distributions are shaped predominantly by botanical origin rather than by extensive surface-bound mineral accumulation.

### Mineral Contents of MP Samples and Recommended Daily Allowance RDA (%) for Female and Male Adults

Bee pollen has long been known as a natural source of essential nutrients, distinguished by its considerable macro- and micro-element content. More recently, interest in pollen has extended beyond nutrition, as multiple studies suggest that it can serve as a sensitive biological indicator for environmental pollution. This indicator capacity is primarily related to its tendency to accumulate elements with respect to their abundance in air, soil, and water. It makes them biological indicators capturing a chemical “fingerprint” of the ecosystem visited by foraging honeybees. The accumulating evidence therefore supports its use as a bioindicator for multi-matrix contamination assessments, positioning bee pollen at the interface of environmental analytics and food science [38, 60, 61].

Potentially toxic elements (PTEs), including arsenic (As), cadmium (Cd), lead (Pb), and nickel (Ni) are ubiquitous in environmental media. Their concentrations fluctuate markedly, reflecting not only natural geochemical baselines but also the spatial footprint of anthropogenic emissions, industrial discharge, and agricultural inputs. As a result, exposure levels often vary widely between regions and ecosystems, even within relatively close geographical boundaries. Several investigations emphasize that the elemental composition of pollen can mirror this variability, effectively tracing the contamination background of honeybee foraging zones [47, 62–64]. Since pollen-derived PTEs may ultimately enter the human food chain-either directly or through processed bee products, systematic exposure evaluation remains a public-health priority. This is particularly relevant for sensitive groups where age-dependent or chronic exposure outcomes may diverge [65–67].

The biological uptake of PTEs into tissues has been associated with oxidative imbalance, inflammation, and systemic toxicity, which in turn may elevate both carcinogenic and non-carcinogenic long-term health risks [68–70]. The extent of these effects varies based on dose, duration of exposure, and individual susceptibility; however, their capacity to create a cumulative toxic load indicates the necessity for ongoing monitoring and preventative dietary exposure modeling [71, 72].

From a nutritional viewpoint, pollen has also been reported to contribute appreciably to recommended daily intake ratios of several metabolically critical minerals such as phosphorus (P), magnesium (Mg), calcium (Ca), copper (Cu), iron (Fe), zinc (Zn), manganese (Mn), selenium (Se), and chromium (Cr) especially when expressed on a dry-weight basis. These findings have encouraged the incorporation of pollen into functional food formulations and micronutrient-supporting supplements [3, 21].

In the present work, monofloral pollen samples were profiled to capture both surface-accessible and bulk-representative elemental composition, using a complementary surface-to-bulk analytical strategy. The results showed a similar concentration gradient across samples (mg/kg), with magnesium having the greatest mean level, followed by iron and calcium. The whole order from highest to lowest was Mg (1434.3) > Fe (294.1) > Ca (172.1) > Zn (56.84) > Mn (52.89) > Na (50.0) > Cu (16.58) > Cr (4.391) > Pb (0.410) > Cd (0.196) > Se (0.0431) > As (0.0345). This hierarchy may represent the physiological functions of these components in plant-derived pollen and their varying absorption or retention characteristics in honeybee-collected biomass. Elemental enrichment analysis revealed that Mn, Fe, and Cu were considerably elevated compared to other observed elemental subsets (*p* < 0.05). Even though enrichment does not always mean that bioavailability is the same for all minerals, the fact that certain elements are more common is interesting since they play a big role in metalloenzyme activity, erythropoiesis, and redox-defense pathways. These factors combined support the claim that monofloral pollen may serve as a valuable resource for micronutrient-targeted supplementation, particularly in instances of nutritional deficiencies associated with Fe, Mn, or Cu. To put the importance of dietary minerals in context, Table 3 shows the RDA contributions that were calculated. The lower absolute mg/kg results seen here compared to the literature can be explained by a moisture-basis analytical correction that was used during data processing. This correction reduced the values by almost 12 times. The difference is important since most published datasets utilize fully dried pollen, while the samples in this study were made from fresh pollen before normalization. Because fresh pollen has a naturally high moisture content, it has lower apparent concentrations until they are stated on a corrected dry-weight basis.

Food is deemed a significant source of minerals if it comprises 15% of the nutritional reference values per 100 g [73]. From this perspective, an adult deficient in certain elements who ingests 40 g of pollen daily will find that pollen is an exceptionally abundant source of certain minerals, as indicated in Table 4. In this context, samples MP1, MP3, MP7, MP9, and MP10 were notable because they had higher levels of Fe and Mn than the others. This suggests that they could be useful for selective mineral support. On the other hand, chromium was much higher in most samples, except for MP2, MP5, MP7, and MP10. This shows that trace-element heterogeneity is very high, even in samples that come from the same area. Taken together, the findings highlight that pollen exhibits marked elemental variability, reinforcing the importance of source-resolved analytics, moisture-adjusted reporting, and mineral-specific nutritional interpretation when evaluating its function as both a dietary supplement candidate and an environmental contamination indicator.

**Table 4:** Contributions of 40 g daily intake of monofloral pollen samples to the recommended daily allowance (RDA) as a percentage (%)

Contributions to RDA (%) values											
Elements	RDA/Al/UL	MP1	MP2	MP3	MP4	MP5	MP6	MP7	MP8	MP9	MP10
Mn	2.3 mg*	9.2	3.6	7.6	5.1	2.3	5.1	7.6	4.2	16.6	16.8
	1.8 mg**	11.8	4.6	9.8	6.6	2.9	6.5	9.7	5.4	21.2	21.5
Fe	8 mg*	23.2	9.3	19.4	8.8	4.9	10.9	15.2	7.9	14.4	11.0
	18 mg**	10.3	4.1	8.6	3.9	2.2	4.8	6.8	3.5	6.4	4.9
Zn	11 mg*	2.7	1.3	1.9	1.8	1.7	1.5	1.6	1.5	1.7	1.9
	8 mg**	3.7	1.8	2.7	2.5	2.3	2.0	2.2	2.1	2.3	2.6
Mg	420 mg*	0.5	0.5	1.1	0.8	2.4	1.4	0.8	0.7	1.0	1.1
	320 mg**	0.6	0.6	1.4	1.0	3.2	1.9	1.1	0.9	1.3	1.4
Cr	0.035 mg*	58.3	6.3	53.9	28.0	0.0	21.9	0.0	24.6	105.6	0.0
	0.025 mg**	81.6	8.9	75.4	39.3	0.0	30.7	0.0	34.5	147.9	0.0
Ca	1000 mg***	0.1	0.1	0.0	0.1	0.0	0.1	0.0	0.1	0.1	0.1
Na	1500 mg***	0.01	0.01	0.01	0.02	0.01	0.01	0.02	0.02	0.01	0.01
Se	0.055 mg***	0.2	0.1	0.2	0.3	0.3	0.4	0.4	0.3	0.2	0.2
Cu	0.9 mg***	7.2	3.5	5.8	6.4	4.9	3.7	5.2	5.7	4.4	3.8

*Male

**Female

***Male and Female

### Evaluation of THQ and CR Values in MP Samples

The exposure of humans to hazardous substances through the consumption of contaminated food matrices has been extensively documented, and scholarly interest in this area has increased in recent years, emphasizing its growing significance for both food safety and environmental health sciences [46, 74–80]. In this context, the current study employs a systematic and health risk–focused analytical methodology to assess the potential negative effects linked to pollen consumption. Risks were specifically quantified using internationally recognized exposure and toxicity indicators such as the Target Hazard Quotient (THQ), Hazard Index (HI), and Target Cancer Risk (CR) derived from potentially harmful substances and pollutants identified in monofloral pollen samples. This study aims to offer a more precise evidence-based understanding of the health effects associated with the consumption of fresh monofloral pollen by combining nutritional context with both non-carcinogenic and carcinogenic risk modeling. In doing so, the study adds to the growing conversation about how to measure dietary exposure, use bioindicators in the environment, and protect public health in ways that are sensitive to age. The individual body weight and pollen intake affect daily exposure to PTEs in both children and adults. Pollen may include evidence of these PTEs as a bioindicator due to their accumulation influenced by environmental factors. In the literature, the recommended daily consumption of bee pollen is generally reported to be approximately 40 g for adults and 20 g for children. These intake levels appear relatively high when considered in the context of dry pollen consumption. Indeed, if exposure and health risk assessments are conducted assuming dry pollen intake at these amounts, the resulting estimates tend to yield substantially elevated toxicity indicators. Therefore, in the present study, all calculations were performed under the assumption of fresh (wet) pollen consumption, which is considered more representative of realistic dietary practices and provides a more conservative and physiologically relevant exposure scenario [30]. According to the EPA’s 2004 criteria, the “reference dose” (RfD) represents an estimate of daily exposure for the human population, including vulnerable subgroups, with the minimal likelihood of adverse effects over a lifetime [81].

Consequently, more assessments employing THQ, HI, and CR are essential. The THQ represents the ratio of exposure to a hazardous substance compared to the reference dose, which indicates the maximum amount considered safe and free from anticipated adverse health effects. Each trace element has its own reference dose. The THQ measure is used to figure out how dangerous a certain substance is to your health in a way that doesn’t cause cancer. If the THQ is less than 1, it means that the exposure is not likely to cause any health problems that are not related to cancer. However, adverse health effects may occur if the THQ level surpasses 1. A THQ value exceeding 1 does not indicate a statistically significant likelihood of non-cancerous adverse health effects; instead, it denotes an elevated risk potential [77]. Therefore, it is imperative to oversee and control exposure levels below the reference dose to alleviate health risks associated with hazardous substances. The research results showed that the Target Hazard Quotient (THQ) values for all the components that were looked at stayed below 1 (THQ < 1; HI < 1). This means that people who are exposed to these substances are not in danger. (US-EPA 2002, 2016) Table 5 shows that all the pollen samples we looked at in our study are safe to eat. According to statistics from the U.S. Environmental Protection Agency US-EPA (2004) safety criteria for carcinogenic risk were established at 1 × 10 − 6 or lower [45, 82].

**Table 5:** Target hazard quotient (THQ) and Hazard Index (HI) values of PTEs in monoflral pollen samples

		THQ for PTE									
Samples	Age Group	Mn	Fe	Zn	Cr	Pb	Cd	Ni	As	Cu	HI
MP1	A	0.020742	0.036327	0.013582	0.079859	1.16E-09	0.016641	2.33E-09	0.027494	0.00725	0.202
	Ch	0.048398	0.084762	0.033051	0.186338	2.72E-06	0.038828	5.43E-09	0.064153	0.016916	0.472
MP2	A	0.008111	0.014509	0.006505	0.008686	1.16E-09	0.007052	2.33E-09	0.013252	0.003563	0.062
	Ch	0.018926	0.033853	0.015829	0.020267	2.72E-06	0.016454	5.43E-09	0.030921	0.008313	0.145
MP3	A	0.017184	0.030374	0.009749	0.073778	1.16E-09	0.017167	2.33E-09	0.022026	0.007209	0.177
	Ch	0.040097	0.070872	0.023724	0.172149	2.72E-06	0.040055	5.43E-09	0.051394	0.016821	0.415
MP4	A	0.011564	0.013715	0.009152	0.038417	0.004716	0.007904	2.33E-09	0.024513	0.006378	0.116
	Ch	0.026982	0.032001	0.022269	0.089639	0.011003	0.018443	5.43E-09	0.057197	0.014883	0.272
MP5	A	0.005084	0.007671	0.008337	1.33E-08	1.16E-09	0.001657	2.33E-09	0.018746	0.003225	0.045
	Ch	0.011862	0.017899	0.020287	3.11E-08	2.72E-06	0.003867	5.43E-09	0.043740	0.007525	0.105
MP6	A	0.011508	0.016998	0.007484	0.030046	1.16E-09	0.016818	2.33E-09	0.014043	0.003654	0.101
	Ch	0.026851	0.039662	0.018211	0.070107	2.72E-06	0.039243	5.43E-09	0.032767	0.008517	0.235
MP7	A	0.017028	0.023836	0.007921	1.33E-08	1.16E-09	0.007899	2.33E-09	0.019939	0.008734	0.085
	Ch	0.022041	0.028846	0.018527	0.078728	2.72E-06	0.016826	5.43E-09	0.051032	0.013411	0.229
MP8	A	0.009446	0.012362	0.007614	0.033742	1.16E-09	0.007211	2.33E-09	0.021871	0.005747	0.098
	Ch	0.087149	0.052765	0.020604	0.337681	2.72E-06	0.015153	5.43E-09	0.038787	0.008489	0.561
MP9	A	0.037352	0.022614	0.008467	0.144721	1.16E-09	0.006494	2.33E-09	0.016623	0.003638	0.240
	Ch	0.087149	0.052765	0.020604	0.337681	2.72E-06	0.015153	5.43E-09	0.038787	0.008489	0.561
MP10	A	0.037901	0.017237	0.009451	1.33E-08	1.16E-09	0.002398	2.33E-09	0.014463	0.004224	0.086
	Ch	0.088450	0.040220	0.022994	3.11E-08	2.72E-06	0.005595	5.43E-09	0.033746	0.009857	0.201

A: Adult

Ch: Child

Results ranging from 1 × 10^− 6^ to 1 × 10^− 4^ are categorized as intermediate risk, while remaining within an acceptable range (US-EPA, 1991, 2004.) The fundamental threshold for human carcinogenicity is regarded as 1 × 10^− 4^. If this threshold is surpassed, the implications must be meticulously assessed regarding public health issues [46]. CR analysis revealed that all pollen samples were safe in terms of Pb, Cd, Ni, and Cd concentrations. However, 6 samples were found to be at moderate risk in terms of Cr concentrations for adults (10^− 4^< CR < 10^− 6^) (Table 6). Among children, two of these six values are perceived as hazardous.

**Table 6:** Carcinogenic Risks of some potentially toxic elements (PTEs) in monoflral pollen samples

CR values for PTE						
Samples	Age Group	Cr	Pb	Cd	Ni	As
MP1	A	5.19E-05	1.47E-14	2.35E-06	2.94E-11	1.21E-06
	Ch	1.21E-04	3.43E-11	5.48E-06	6.86E-11	2.83E-06
MP2	A	5.65E-06	1.47E-14	9.95E-07	2.94E-11	5.95E-07
	Ch	1.32E-05	3.43E-11	2.32E-06	6.86E-11	1.39E-06
MP3	A	4.80E-05	1.47E-14	2.42E-06	2.94E-11	1.20E-06
	Ch	1.12E-04	3.43E-11	5.65E-06	6.86E-11	2.81E-06
MP4	A	2.50E-05	5.96E-08	1.12E-06	2.94E-11	1.07E-06
	Ch	5.83E-05	1.39E-07	2.60E-06	6.86E-11	2.49E-06
MP5	A	8.65E-12	1.47E-14	2.34E-07	2.94E-11	5.39E-07
	Ch	2.02E-11	3.43E-11	5.46E-07	6.86E-11	1.26E-06
MP6	A	1.95E-05	1.47E-14	2.37E-06	2.94E-11	6.10E-07
	Ch	4.56E-05	3.43E-11	5.54E-06	6.86E-11	1.42E-06
MP7	A	8.65E-12	1.47E-14	1.11E-06	2.94E-11	1.46E-06
	Ch	2.02E-11	3.43E-11	2.60E-06	6.86E-11	3.41E-06
MP8	A	2.19E-05	1.47E-14	1.02E-06	2.94E-11	9.61E-07
	Ch	5.12E-05	3.43E-11	2.37E-06	6.86E-11	2.24E-06
MP9	A	9.41E-05	1.47E-14	9.17E-07	2.94E-11	6.08E-07
	Ch	2.19E-04	3.43E-11	2.14E-06	6.86E-11	1.42E-06
MP10	A	8.65E-12	1.47E-14	3.38E-07	2.94E-11	7.06E-07
	Ch	2.02E-11	3.43E-11	7.90E-07	6.86E-11	1.65E-06

Tutun, Aluç [37] determined non-carcinogenic toxicity values in pollen and propolis samples collected from the western districts of Türkiye. The yearly per capita pollen consumption in this study was 8.45 g, indicating that the results were quite modest and devoid of risk. This value was taken as the average annual per capita consumption for Türkiye.

In another study conducted in 2023, pollen samples from northeastern Türkiye were analyzed, and the results were used to calculate both non-carcinogenic and carcinogenic toxicity. This study was the first in literature to assess the carcinogenic risk of pollen [36]. The study posited that the consumer consistently ingested 40 g of pollen each day. The findings of this study indicate that lead levels were, on average, lower than those observed in the last study; however, arsenic and nickel levels were significantly elevated compared to previous research. This study, like the preceding one, concentrated on the examination of polyfloral pollen samples. This study examined the identical mineral elements as ours and produced notably comparable average results. In comparison to this investigation, monofloral pollen samples had a greater concentration of Fe.

In terms of potentially toxic elements, HI levels in polyfloral pollen range from 0.31 to 0.65, but monofloral pollen has lower average levels, ranging from 0.045 to 0.561. The primary distinction is in the ingredients that present carcinogenic risks. The outcomes in polyfloral samples are deemed moderate and hazardous for Cr, As, and Ni, whereas the polyfloral samples seem to pose little risk. Kostić, Dojčinović [59] conducted one of the earliest health risk assessments on monofloral pollen, utilizing sunflower (Helianthus annuus) as a model matrix. They reported that while THQ values for arsenic (As) remained below 1 in adults indicating no significant non-carcinogenic risk under long-term exposure assumptions, the estimated risk for younger populations exceeded the safety threshold of 1, indicating a potential age-dependent vulnerability. In a geographically stratified Turkish study, Temizer [3] analyzed pollen samples from western and eastern urban centers of Türkiye, revealing THQ values consistently below 1, indicating a lack of significant non-carcinogenic risk. Likewise, commercial pollen products assessed in Türkiye by Sevin, Tutun [11] indicated no significant non-carcinogenic or carcinogenic risk values, thereby corroborating their findings with recognized dietary exposure safety thresholds. The research findings collectively emphasize that while pollen is typically regarded as a low-risk food matrix for adults, exposure-based risk metrics may differ significantly among various age groups and environmental foraging regions, necessitating ongoing age- and source-sensitive monitoring in future evaluations.

### Multivariate Profiling and Statistical Differentiation of Elemental Composition

**Fig. 4:**
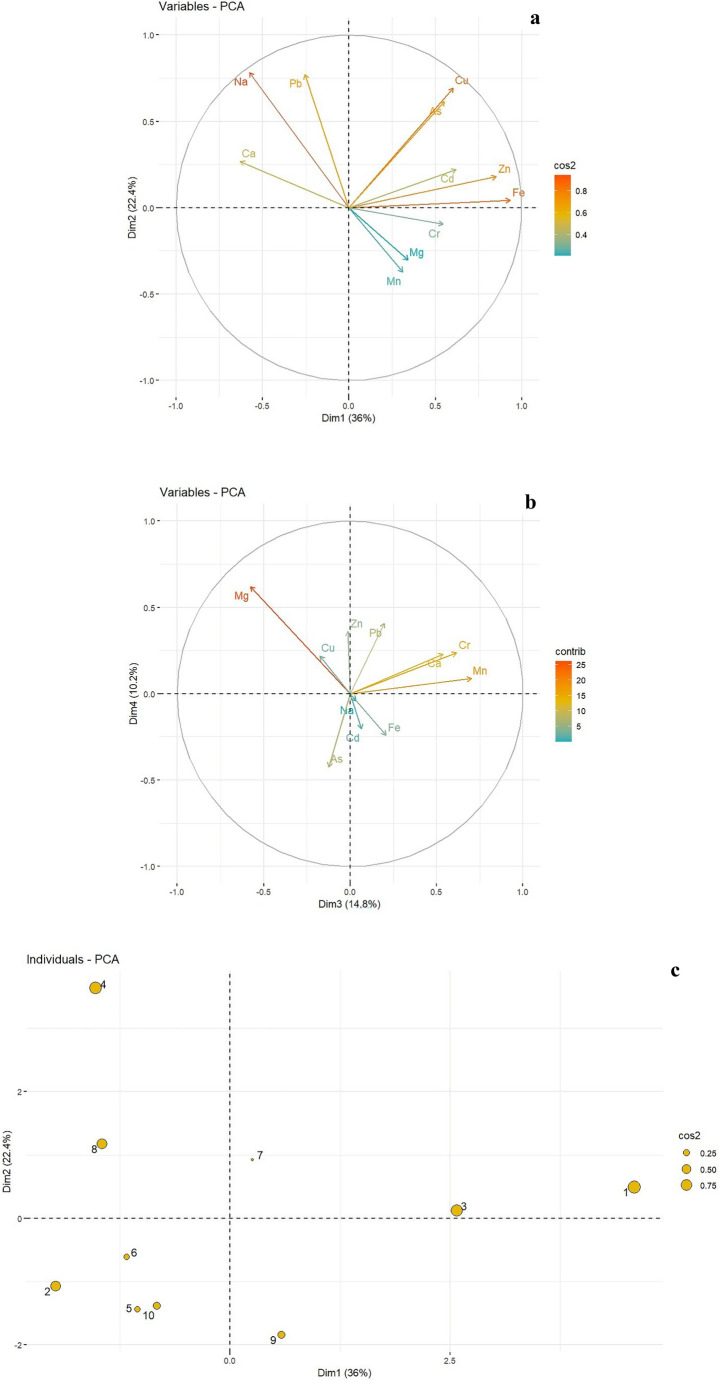
Principal component analysis (PCA) of variables. **a** PC1 and PC2 map, (**b**) PC2 and PC3 map for monofloral pollen samples

**Fig. 5:**
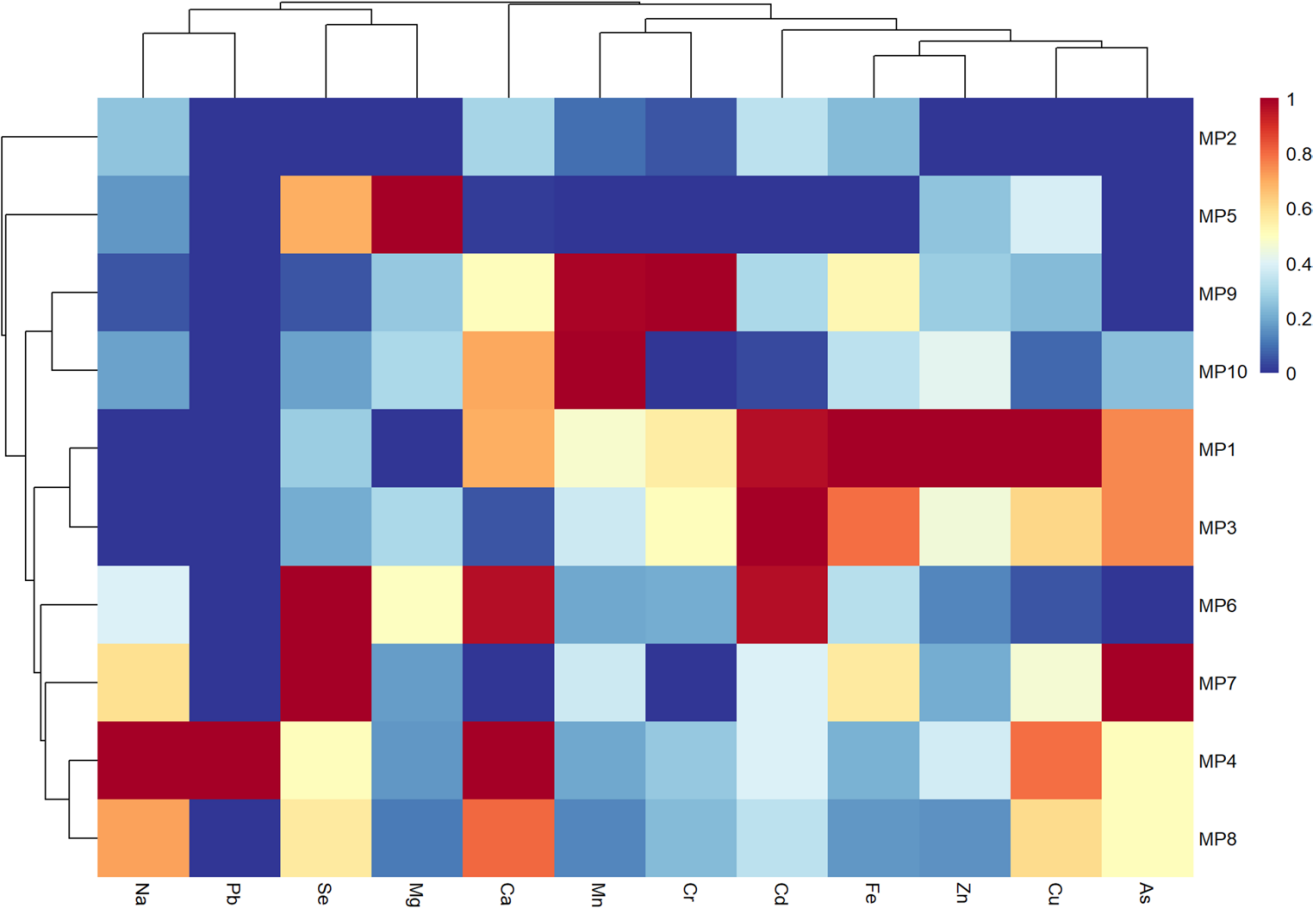
Heatmap-based elemental fingerprinting of monofloral pollen varieties

The PCA results in Fig. 4 indicate that the first four principal components (eigenvalues > 1) explain 83.1% of the total variance, demonstrating that a small number of components capture most of the compositional variability among monofloral pollen sources. The eigenvalues of PC1–PC4 (4.0, 2.5, 1.6, and 1.1) correspond to variance contributions of 36.0%, 22.4%, 14.8%, and 10.2%, respectively, confirming strong multivariate structure in the dataset. Dimension-based variable loading patterns reveal that Fe, Zn, and Ca are the most influential elements in Dim1/PC1, whereas Na, Pb, and Cu dominate Dim2/PC2, suggesting that macro-elemental accumulation and trace metal presence drive the largest axes of differentiation. The subsequent components highlight secondary contrasts shaped by Mn, Cr, Mg, and As distributions, indicating that micronutrient and PTE variability remains source-dependent even when absolute concentrations are low. The individual PCA plot further shows that MP1 and MP4 are spatially separated from the main pollen clusters, reflecting their distinctive elemental proportions relative to other sources. Hierarchical clustering (Bray–Curtis/UPGMA) supports this multivariate separation, grouping samples into three primary compositional clusters: (i) MP2, MP5, MP9, and MP10, characterized by ultra-trace PTE levels and relatively high Mg in MP5; (ii) MP1 and MP3, displaying comparatively higher micronutrient and transition-metal content (Mn, Fe, Zn, Cu, Cd); and (iii) MP6, MP7, MP4, and MP8, distinguished by elevated Na and As, with Na most pronounced in MP4 and MP8, and As peaking in MP7. Although Pb occurs in all samples at low or trace levels, a distinct increase is observed only in MP4, as visualized in the normalized heatmap (Fig. 5).

Although the PCA and ANOVA results demonstrate pronounced heterogeneity in the distribution of macro- and microelements among monofloral pollen samples, this variability does not translate into a strict clustering according to botanical origin. This indicates that, while monoflorality ensures taxonomic purity of pollen grains, elemental composition reflects the combined influence of plant physiological uptake, local soil geochemistry, atmospheric deposition, and post-collection processes rather than a single taxon-driven mechanism.

Botanical origin alone cannot explain pollen mineral composition; geographical and environmental factors significantly influence mineral content independently. The evidence is substantial and specific. Lilek, Kandolf Borovšak [57] found that mineral composition “varies greatly depending on botanical and geographical origin,” indicating dual influences. More precisely, Pavlin, Kočar [83] demonstrated that certain elements (Mn, K, Ca) are “primarily influenced by the type of pollen present,” while “levels of Na, Mg, and Fe were found to be more strongly influenced by environmental factors.” This element-specific distinction is critical: it shows that even within a single botanical source, environmental conditions alter mineral profiles. Supporting this, de Oliveira, de Abreu [84] found statistically significant differences in mineral content between geographical locations and attributed these to “geogenic and anthropic contributions of the sites.” Collectively, these three studies across different bee pollen systems demonstrate that geographical origin and environmental factors operate as independent variables from botanical origin in determining final mineral composition. Supporting this, Yang, Wu [23] analyzed 12 monofloral varieties and reported “some significant differences between samples” in mineral elements including K, Ca, Mg, Zn, Fe, and Mn. Additionally, Modro, Silva [85] found that unifloral pollen samples (> 80% single pollen type) exhibited “protein content variation,” indicating compositional heterogeneity within monofloral batches. Lukšić, Mucalo [86] examined pollen grains from ten individuals of the same species (Vitis sylvestris). They found that botanical origin alone does not determine elemental uniformity; geographical location, soil composition, and individual plant genetics all contribute to elemental variation in monofloral pollen. These findings collectively establish that botanical monoflorality does not guarantee elemental uniformity.

Consequently, the multivariate separation observed in PCA represents a composite environmental–biological signal rather than direct botanical control. This finding supports the interpretation of bee pollen as a sensitive integrator of both biological and environmental factors, rather than a matrix in which elemental variability can be attributed exclusively to the predominant floral source.

The statistical analyses show that elemental heterogeneity is not random but structured, and partial correlations with predominant plant species are evident for specific element groups rather than across the entire elemental profile. ANOVA results indicate that several macro- and microelements differ significantly among monofloral pollen types, while PCA and heatmap analyses reveal sample groupings driven by subsets of elements (e.g., Mg, Fe, Mn, Zn) that are known to be influenced by plant physiological uptake and allocation processes. However, this taxon-related signal is superimposed on strong environmental contributions, resulting in multivariate patterns where botanical origin contributes to, but does not solely determine, the overall elemental distribution observed in pollen samples. Plant species do influence element uptake and transfer to pollen, though direct evidence specific to pollen transfer remains limited. The available evidence shows mixed specificity. Djubina, Jansone [56] directly examined pollen from hazel and alder, finding that “the concentration of these elements may vary depending on factors such as plant species and growth conditions,” with hazel pollen containing lead, zinc, and tin while alder showed different elemental profiles. However, this represents only two species with preliminary findings. More robust evidence comes from Cloutier-Hurteau, Gauthier [87], who traced nine trace elements (Ba, Cd, Cr, Cu, Mn, Ni, Pb, Tl, Zn) from soil through roots to ragweed pollen across 26 urban sites. They found significant predictive models for cadmium, nickel, and lead transfer to pollen, though not for all elements tested. Broader support exists: Peura, Saetre [88] and Subramanian, Subha [89] confirm that “elemental concentrations in plants are expected to vary with plant species,” though these studies focused on general plant tissues rather than pollen specifically. The evidence suggests species influence element transfer to pollen, but pollen-specific research remains sparse.

## Conclusion

This study indicated that monofloral bee pollen samples have unique elemental profiles that depend on their source, indicating that botanical origin and local environmental conditions significantly influence mineral uptake and trace-element accumulation. Magnesium, iron, and manganese were recognized as the most distinguishing macro- and micro-elements, indicating their potential use as chemical markers in nutritional classification and environmental contamination monitoring. Non-carcinogenic risk indicators (THQ and HI) were below safety levels (HI < 1) for both adults and children, indicating minimal systemic toxicity under plausible dietary intake scenarios. Carcinogenic-risk modeling showed that chromium and arsenic could exceed tolerable risk thresholds in specific scenarios of kid exposure (CR > 1 × 10⁻⁴), point out age-dependent exposure even at ultra-trace concentrations. From a nutritional perspective, various monofloral pollens provided significant contributions to the recommended dietary allowances for iron and manganese, highlighting pollen’s importance as a helpful micronutrient source for deficiencies. The findings collectively underline that monofloral pollen can consistently aid in nutritional-value classification and PTE exposure interpretation; however, future safety assessments should integrate life-stage sensitivity and long-term source-specific monitoring, especially for younger consumers. Elemental heterogeneity within a common area was verified, and moisture-basis normalization was established as crucial for precise comparison with dry-weight literature standards.

Although this study provides comprehensive information on the total macro- and microelement composition of monofloral bee pollen, it should be emphasized that total elemental concentrations do not directly reflect nutritional bioaccessibility or bioavailability. Given the complex plant-derived matrix of pollen and the presence of compounds that may modulate mineral absorption, future studies incorporating in vitro digestion models and bioavailability assessments are essential to accurately evaluate the nutritional contribution of bee pollen. Such investigations will be critical for substantiating dietary intake claims and supporting the safe and effective use of bee pollen in nutritional applications.

## Data Availability

Data are available from the corresponding author upon reasonable request, in accordance with institutional and ethical data-sharing policies.
